# Evaluation of Ki-67 Expression in Disordered Proliferative Endometrium, Endometrial Hyperplasia, and Endometrial Carcinoma

**DOI:** 10.7759/cureus.107849

**Published:** 2026-04-27

**Authors:** Amrita Chauhan, Surekha U Arakeri, Neelamma Patil

**Affiliations:** 1 Pathology, Shri B M Patil Medical College Hospital and Research Centre, Vijayapura, IND; 2 Obstetrics and Gynecology, Shri B M Patil Medical College Hospital and Research Centre, Vijayapura, IND

**Keywords:** anatomy, endometrium, gynecology, immunohistochemistry, pathology

## Abstract

Introduction: Endometrial hyperplasia (EH) is characterized by elevated estrogen levels, which can lead to increased endometrial proliferation. Unopposed hyperestrogenism and elevated estrogen levels in conditions such as Lynch syndrome and estrogen-producing ovarian tumors can trigger endometrial proliferation and act as a predisposing factor for atypical endometrial hyperplasia (AEH) and endometrial carcinoma (EC). Recently, it has been considered that there is a progression from EH to endometrioid carcinoma. Disordered proliferative endometrium (DPE) is quantitatively similar to EH without atypia. The immunohistochemistry (IHC) marker Ki-67 reflects the degree of proliferation of malignant cells, as well as tumor invasion, metastasis, and prognosis in various malignancies. The aim of the study was to evaluate the expression of Ki-67 in DPE, EH without atypia, AEH, and EC, and to compare Ki-67 expression in low-grade and high-grade EC.

Methodology: A hospital-based cross-sectional study was conducted in the Histopathology Section of the Department of Pathology using endometrial tissue samples obtained by endometrial curettage and hysterectomy from patients diagnosed with DPE, hyperplastic endometrium, and EC. For each case, an IHC study for the Ki-67 marker was performed.

Results: Out of 68 cases of endometrial lesions, 21 (30.88%) were DPE, 13 (19.12%) were EH with atypia, 16 (23.53%) were EH without atypia, and 18 (26.47%) were EC. The mean total Ki-67 positivity in EC was 60.16±24.26; in AEH, 22.56±4.98; in EH without atypia, 7.86±6.21; and in DPE, 13.23±15.68.

Conclusion: Ki-67 can help differentiate DPE from EH without atypia and AEH when diagnostic difficulty arises on Hematoxylin and Eosin (H&E)-stained slides. Ki-67 expression was significantly higher in high-grade ECs than in low-grade ECs, suggesting a role for Ki-67 as a prognostic indicator and biomarker of aggressiveness. Hence, Ki-67 marker studies should be routinely performed in endometrial lesions such as DPE, EH without atypia, and AEH.

## Introduction

The incidence of endometrial carcinoma (EC) is increasing worldwide. It is the fourth most common malignancy in females [1-3]. The risk of developing EC is 3.1%, and the five-year survival rate is 81% [2]. The incidence of EC in India is 4.3 cases per 100000 females [4]. Over the last two decades, clinicopathological, immunohistochemical, and molecular studies have generated substantial data on EC. Based on these data, two major endometrial precursor lesions, atypical endometrial hyperplasia (AEH) and endometrial intraepithelial carcinoma (EIC), have been recognized [3].

Endometrial hyperplasia (EH) is typically associated with a hormonal imbalance characterized by elevated estrogen levels that trigger endometrial proliferation. Unopposed hyperestrogenism is a risk factor for AEH/endometrioid intraepithelial neoplasia (EIN) and EC. Conditions such as early menarche, late menopause, estrogen-producing ovarian tumors, and Lynch syndrome lead to prolonged unopposed estrogen exposure, which acts as a risk factor for EC. Thus, EH is one of the risk factors for EC [5]. Endometrioid carcinoma is often preceded by AEH. Recently, it has been considered that there is progression from EH to endometrioid carcinoma. Hence, EH is regarded as a premalignant lesion [3].

In perimenopausal and postmenopausal women, the disordered proliferative phase (DPP) of the endometrium is more commonly noted. Hormonal imbalance between estrogen and progesterone, with low levels of progesterone and high levels of estrogen, can lead to the development of DPP endometrium. It can also progress to EH and EC [4]. DPP endometrium is quantitatively similar to EH without atypia. However, in DPP, the lesion is focal. DPP is characterized by endometrial glands that are enlarged and irregular in shape. These abnormal glands are interspersed with normal-appearing proliferative glands. The focal nature of the glandular abnormality is the characteristic feature of DPP, which helps differentiate it from EH [6].

The IHC marker Ki-67 plays a crucial role in cell proliferation [7]. Ki-67 expression is significantly higher in atypical hyperplasia of the endometrium compared to EH without atypia [5,8]. Ki-67 provides information on the proliferation rate of tumor cells, tumor invasion, tumor spread, and prognosis of malignant tumors [9].

Ki-67 was found to reduce individual subjective bias in diagnosing EH with atypia and EH without atypia, as Ki-67 showed increased expression in atypical hyperplasia and EC compared with hyperplasia without atypia. Hence, it has been suggested that Ki-67 can be used to evaluate the progression of EH toward AEH and EC [10].

Ki-67 overexpression is associated with tumor cell proliferation in malignant lesions. Hence, Ki-67 expression is used to assess the aggressiveness of malignant tumors. Ki-67 is considered a reliable marker for evaluating the progression of premalignant lesions to malignancy and in determining the aggressiveness of malignancy in breast, lung, prostate, cervical, central nervous system, and uterine cancers [11]. In one study, disease-free survival was higher in patients with EC with a low Ki-67 index than in those with a high Ki-67 index. This may help in the management of adjuvant hormone therapy in patients with high Ki-67 [12]. Hence, this study was undertaken to evaluate Ki-67 expression in disordered proliferative endometrium (DPE), hyperplastic endometrium, and EC.

## Materials and methods

Source of data

The study was conducted in the Histopathology Section of the Department of Pathology on endometrial tissue obtained by endometrial curettage and from hysterectomy specimens of patients diagnosed with DPE, hyperplastic endometrium, and EC. The study period was from March 2024 to October 2025. It was a hospital-based, cross-sectional, observational study. The study comprised three years of retrospective and two years of prospective endometrial tissue samples. Slides and blocks of study cases from 2021 to December 2023 were retrospectively collected, and clinical details were obtained from patients’ records. Hematoxylin and Eosin (H&E)-stained sections were evaluated, representative blocks were selected for the Ki-67 marker study, and a detailed clinical history was obtained. Institutional ethical clearance was obtained before conducting the study (BLDE(DU)/IEC-SBMPMC/133/2023-24).

Inclusion and exclusion criteria

All cases of endometrial curettage and endometrial tissue obtained from hysterectomy specimens diagnosed as DPE, EH without atypia, AEH/EIN, and EC were included. Cases in which the tissue was inadequate for immunohistochemical evaluation were excluded.

Data collection methods

Immunohistochemistry (IHC) for Ki-67 was performed on tissue blocks from cases diagnosed with DPE, hyperplastic endometrium, or EC. Cases in which the tissue was inadequate for immunohistochemical evaluation were excluded. For IHC, tonsillar tissue was used as the positive control. Evaluation of the IHC slides was performed as follows. All nuclei showing Ki-67 staining were counted as positive for Ki-67 expression. In all cases, nuclear positivity on IHC was counted manually. For manual counting, the fields showing maximum nuclear staining were selected. Nuclear positivity in 1000 glandular epithelial cells was counted under high-power magnification (400×). The Ki-67 percentage positivity was calculated as the total number of positive nuclei divided by the total number of cells counted. Additionally, Ki-67 expression was evaluated in cells showing strong or weak positivity [5,9]. Interobserver variability and reproducibility were not evaluated.

Sample size

With an anticipated proportion of Ki-67 immunostaining of 83.3%, the study required 67 patients to achieve 95% confidence and 9% absolute precision [5]. Hence, 68 samples were included in the study.

Statistical analysis was performed using the Statistical Package for the Social Sciences (SPSS, Version 20 (IBM Corp., Armonk, NY, USA)). Descriptive results such as mean ± standard deviation, frequencies, and percentages were presented using appropriate tables and diagrams. For comparisons among more than two independent groups, the Kruskal-Wallis test was used. Associations between categorical variables were analyzed using the Chi-square test. A p-value of less than 0.05 (p<0.05) was considered statistically significant.

## Results

In the present study, 68 cases of endometrial lesions were evaluated for Ki-67 expression. The endometrial lesions included DPE, EH, and EC. Ki-67 evaluation was performed in all study cases, and its correlation was assessed among cases of DPE, EH, and EC. In addition, Ki-67 expression was compared between low-grade and high-grade ECs.

The maximum number of endometrial lesions observed in the study was in the 41 to 50 years age group, accounting for 28 (41.18%), followed by the 51 to 60 years age group, accounting for 15 (22.06%), and the 31 to 40 years age group, accounting for 14 (20.59%). In the 61 to 70 years age group, six (8.82%) cases were studied, and in the 21 to 30 years age group, five (7.35%) cases were noted. The mean age of the study participants was 46.22 years. The youngest patient was 24 years old, and the oldest was 68 years old.

The most common histopathological finding was DPE, accounting for 21 cases (30.88%), followed by EC in 18 cases (26.47%), EH without atypia in 16 cases (23.53%), and AEH in 13 cases (19.12%).

Cases of DPE were most common in the 41 to 50 years age group, accounting for 13 (61.90%). AEH was more commonly observed in the 51 to 60 years age group, amounting to six (46.15%), followed by the 41 to 50 years age group, amounting to four (30.76%). EC was most common in the 51 to 70 years age group, amounting to 13 (72.22%) cases, followed by the 41 to 50 years age group, amounting to four (22.22%).

The most common clinical presentation was menorrhagia, amounting to 31 (45.58%), followed by abnormal uterine bleeding, amounting to 16 (23.53%), and postmenopausal bleeding, amounting to 12 (17.64%). In a few cases, features suggestive of chronic pelvic inflammatory disease and white discharge were noted, amounting to seven (10.29%) and two (2.94%), respectively.

**Figure 1:**
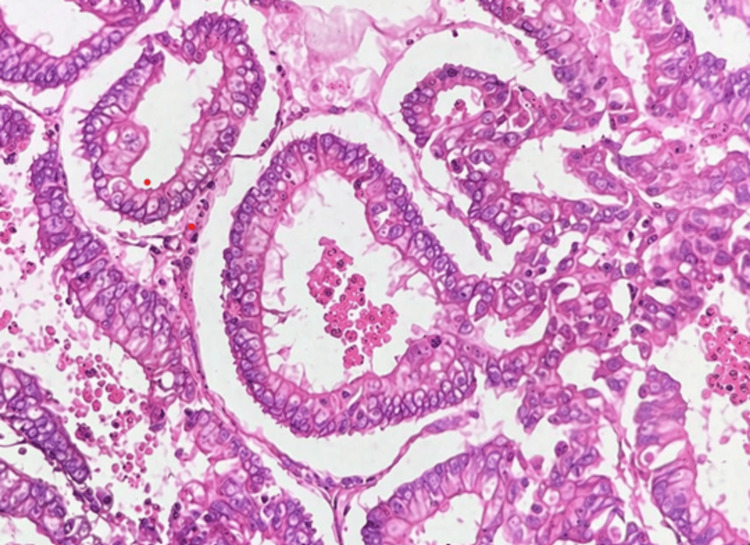
Photomicrograph showing low-grade endometrioid EC (H&E, 400×) H&E: Hematoxylin and Eosin; EC: endometrial carcinoma

**Figure 2:**
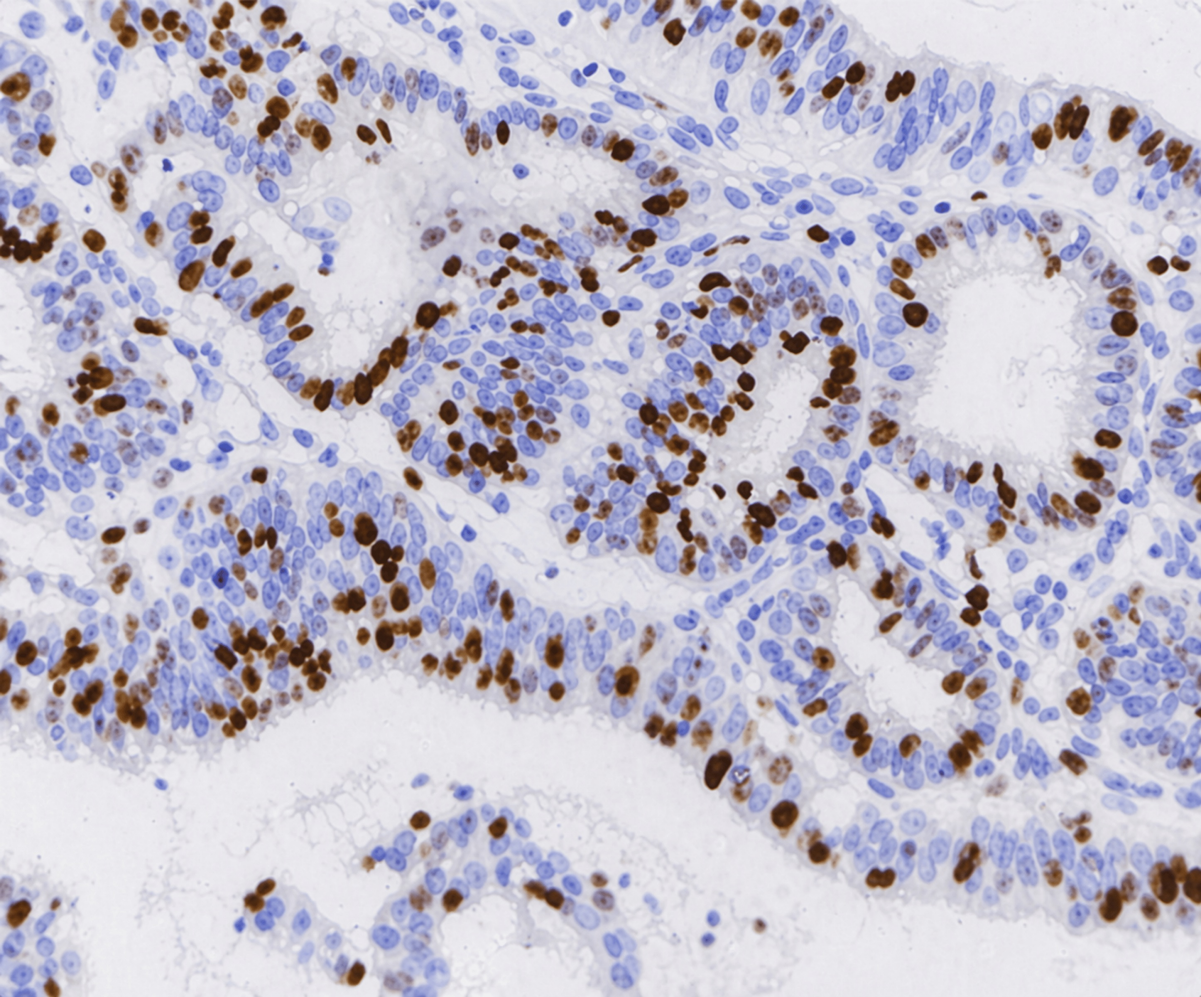
Photomicrograph showing low-grade endometrioid EC (IHC, 400×) IHC: immunohistochemistry; EC: endometrial carcinoma

**Figure 3:**
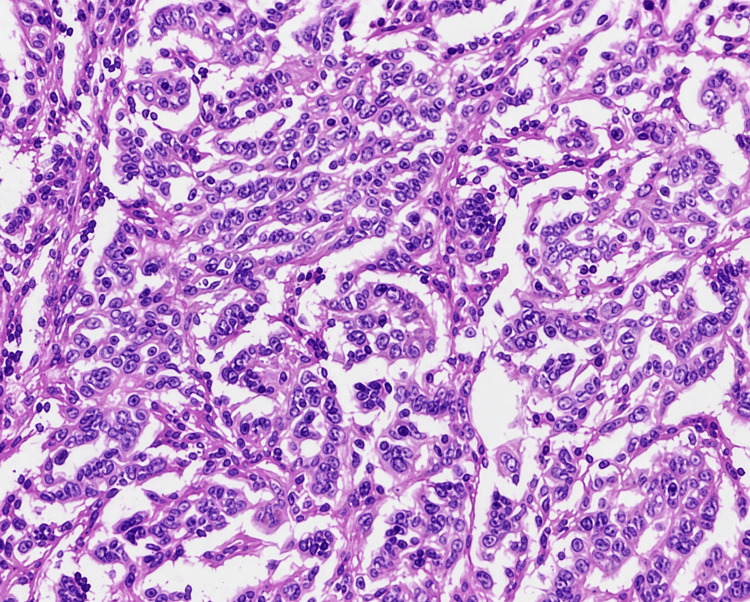
Photomicrograph of high-grade endometrioid EC (H&E, 400×) H&E: Hematoxylin and Eosin; EC: endometrial carcinoma

**Figure 4:**
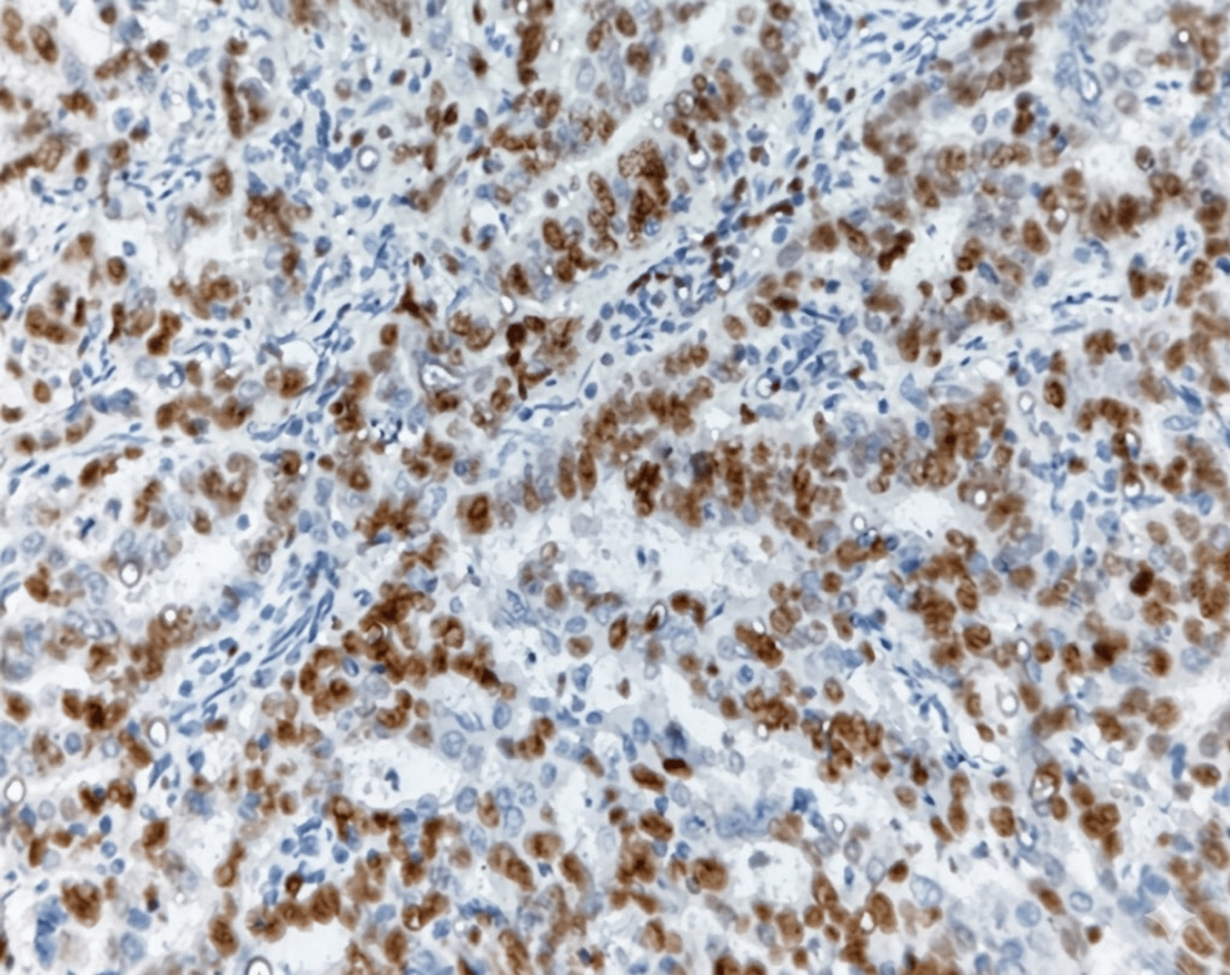
Photomicrograph showing high-grade endometrioid EC (IHC, 400×) IHC: immunohistochemistry; EC: endometrial carcinoma

**Figure 5:**
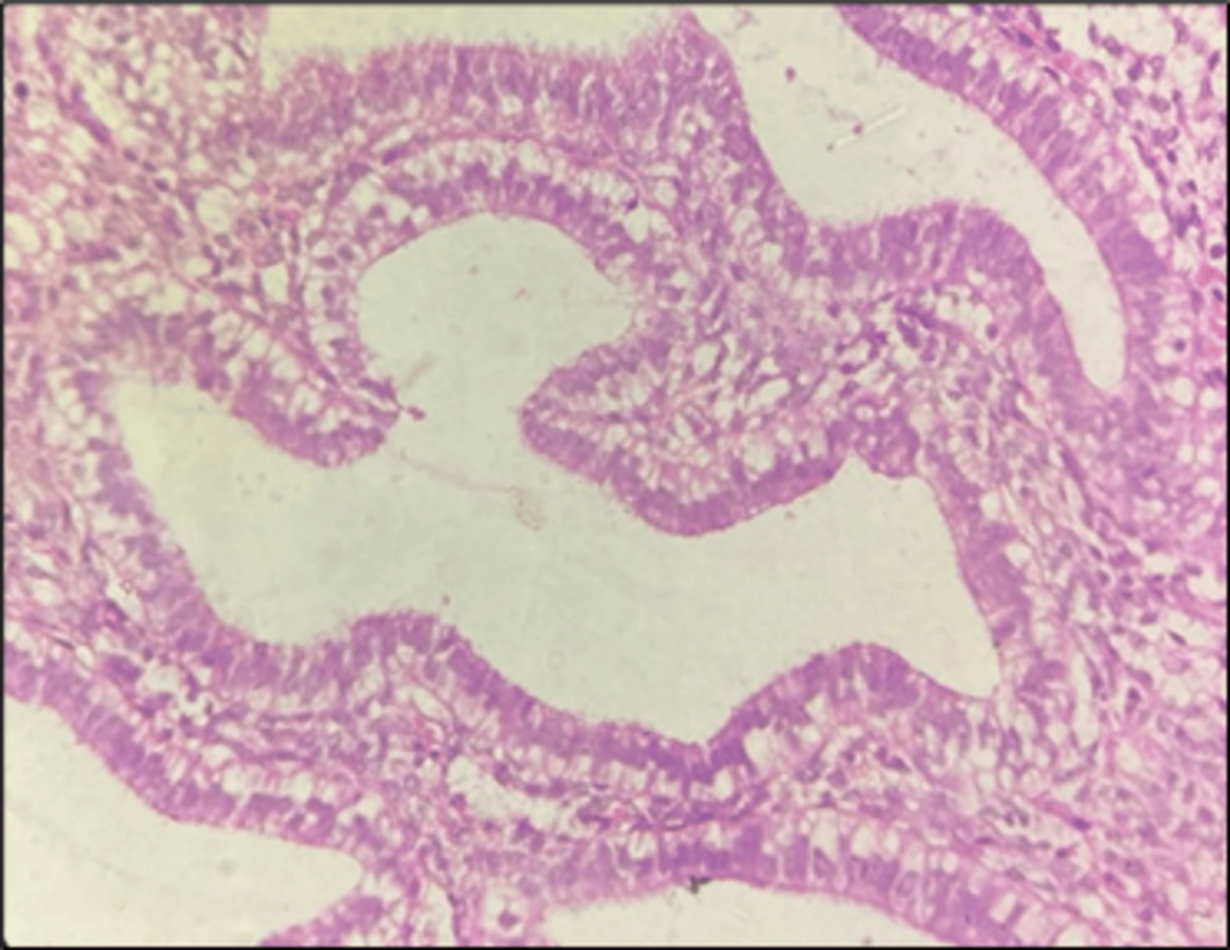
Photomicrograph of AEH (H&E, 400×) H&E: Hematoxylin and Eosin; AEH: atypical endometrial hyperplasia

**Figure 6:**
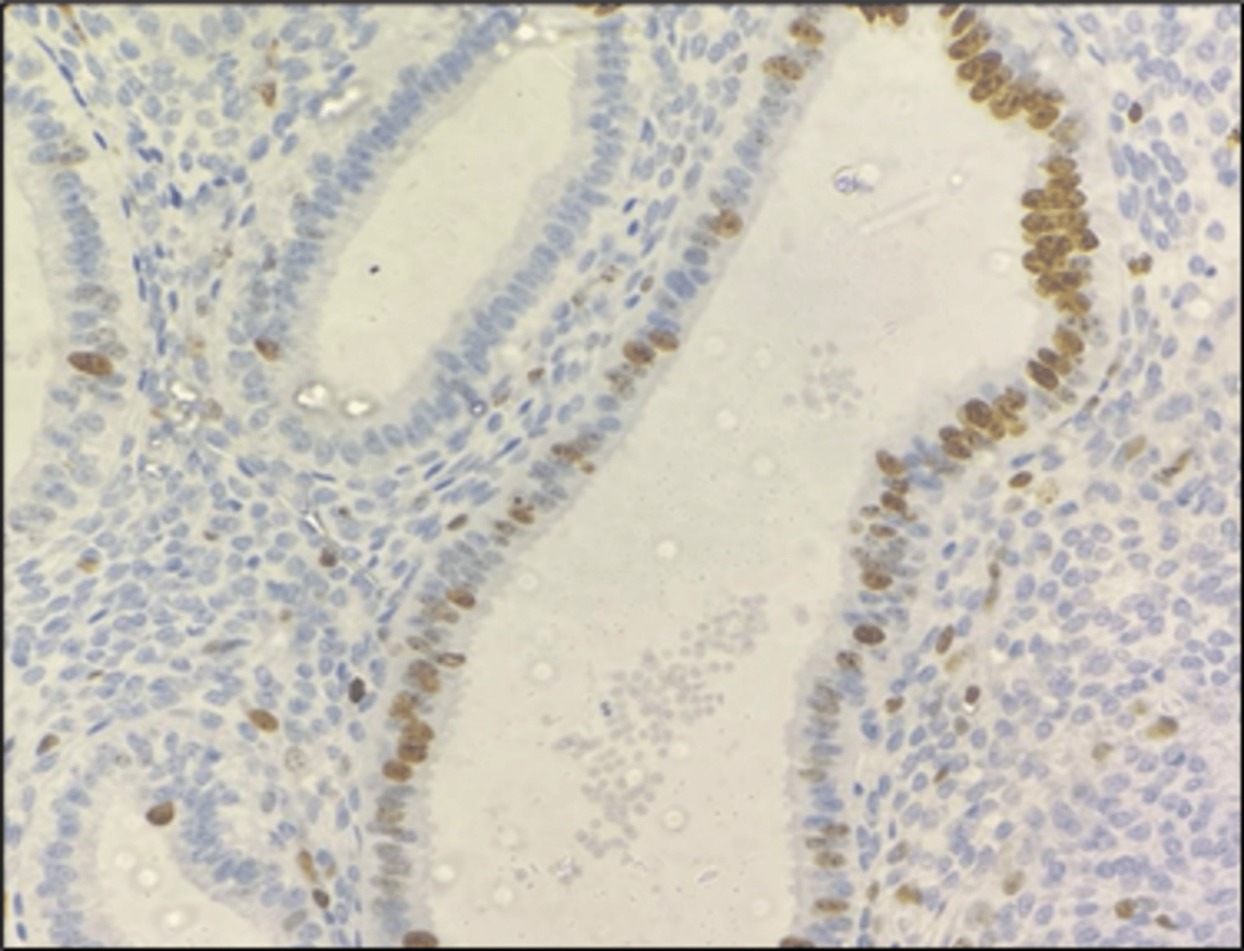
Photomicrograph of AEH (IHC, 400×) IHC: immunohistochemistry; AEH: atypical endometrial hyperplasia

The highest mean total positivity, strong positivity, and weak positivity for Ki-67 expression were observed in EC, followed by AEH (Table 1, Figures 1, 2, 3, 4, 5, 6).

**Table 1:** Mean total positivity, strong positivity, and weak positivity of Ki-67 expression in various endometrial lesions SD: Standard deviation; EH: endometrial hyperplasia; DPE: disordered proliferative endometrium; EC: endometrial carcinoma

Types of endometrial lesions	Number of cases	Total positivity (mean)	SD	Strong positivity (mean)	SD	Weak positivity (mean)	SD
DPE	21	13.23	15.68	10.55	13.79	2.67	4.11
EH without atypia	16	7.86	6.21	6.67	5.60	1.18	0.94
Endometrial atypical hyperplasia	13	22.56	4.98	17.95	5.16	4.71	3.10
EC	18	60.16	24.26	54.34	25.88	5.50	4.74
Total	68	26.17	26.10	22.64	24.76	3.46	3.94

**Figure 7:**
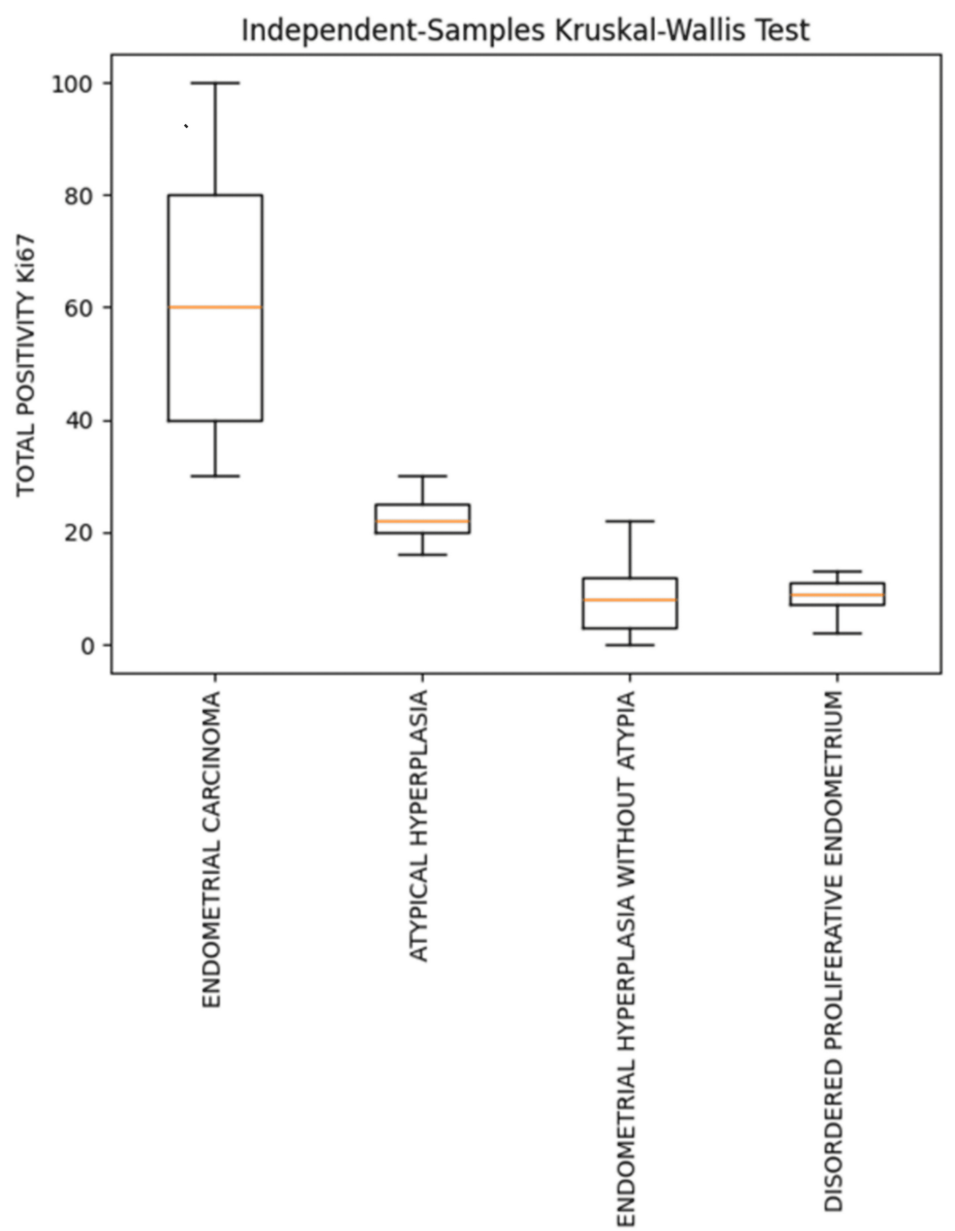
Box plot showing comparison of total Ki-67 positivity in various endometrial lesions

When total Ki-67 positivity was compared across various endometrial lesions, Ki-67 expression was higher in EC than in DPE, with a statistically significant difference (p<0.001). Ki-67 was slightly higher in EH than in DPE; however, the difference was not statistically significant (Table 2, Figure 7).

**Table 2:** Pairwise comparison of Ki-67 positivity in various endometrial lesions (post hoc test) ^*^P-value <0.05 is considered significant. EH: endometrial hyperplasia; DPE: disordered proliferative endometrium; EC: endometrial carcinoma

Types of endometrial lesions		Total positivity		Strong positivity		Weak positivity	
		Test statistics (post hoc test)	P- value	Test statistics (post hoc test)	P- value	Test statistics (post hoc test)	P- value
EH without atypia	DPE	-5.13	0.434	-3.51	0.59	-9.93	0.13
DPE	EC	35.08	0.00^*^	35.51	0.000^*^	15.86	0.012^*^
EH without atypia	Endometrial atypical hyperplasia	22.16	0.003^*^	20.20	0.006^*^	25.21	0.001^*^
EH without atypia	EC	40.21	0.00^*^	39.03	0.000^*^	25.80	0.000^*^
DPE	Endometrial atypical hyperplasia	17.02	0.01^*^	16.68	0.017^*^	15.28	0.028^*^
Endometrial atypical hyperplasia	EC	18.05	0.01^*^	18.82	0.009^*^	0.58	0.93

**Figure 8:**
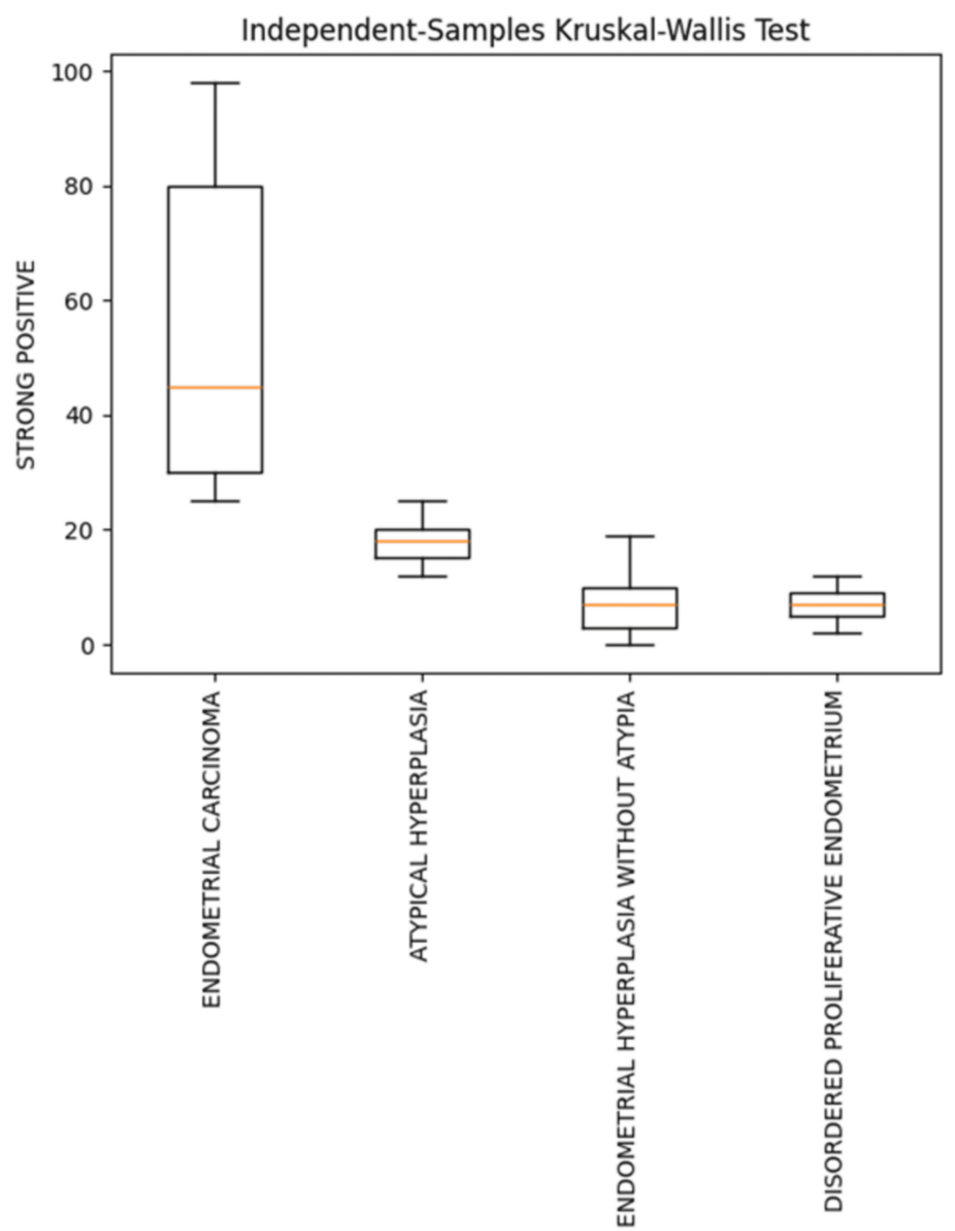
Box plot showing comparison of strong Ki-67 positivity in various endometrial lesions

When strong Ki-67 positivity was compared across different endometrial lesions, a statistically significant difference was observed between DPE and EC (p<0.001). Similarly, the comparison between EH and EC showed a statistically significant difference in Ki-67 expression (Table 2, Figure 8).

**Figure 9:**
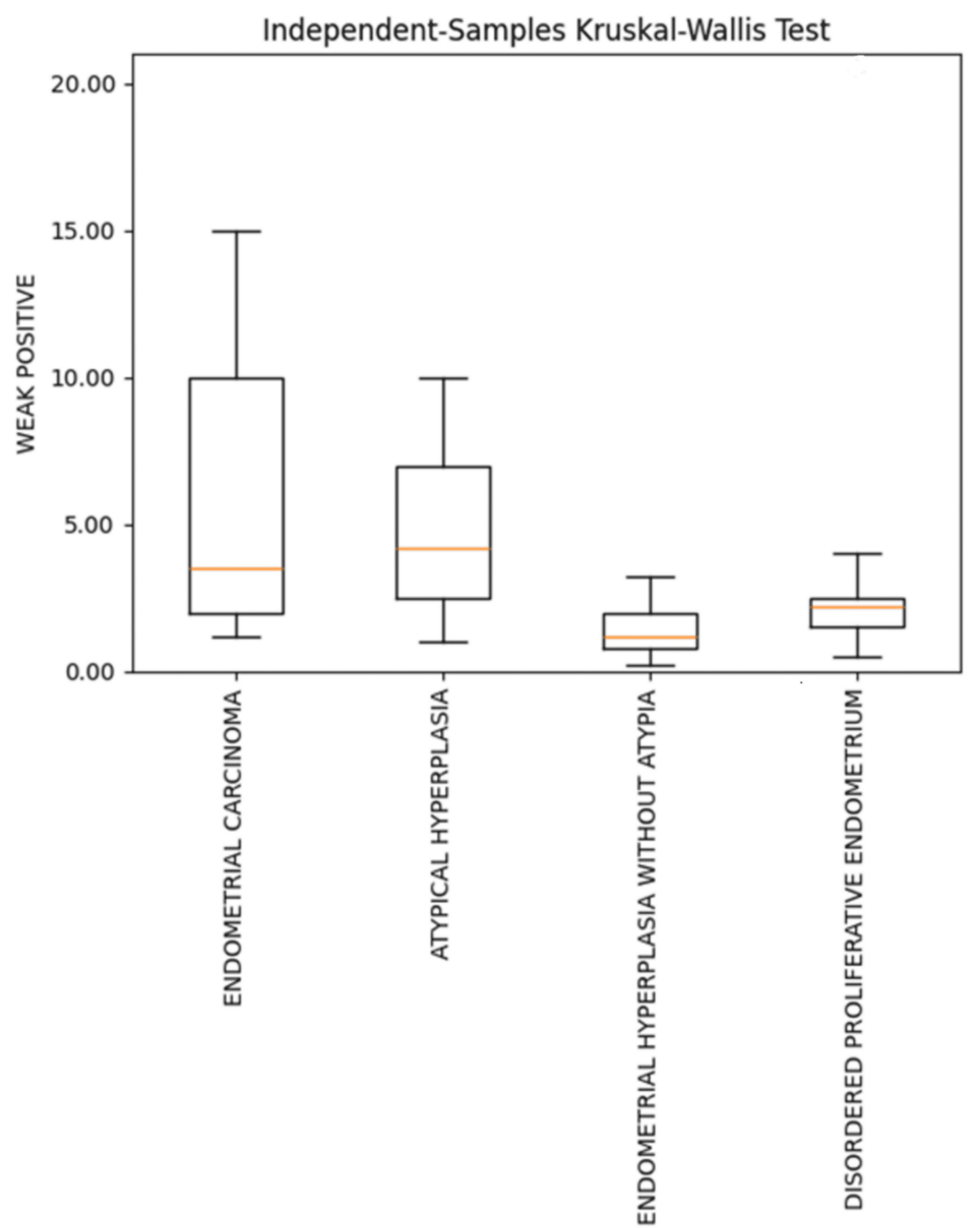
Box plot showing comparison of weak Ki-67 positivity in various endometrial lesions

When weak Ki-67 positivity was compared among different endometrial lesions, a statistically significant difference was observed between DPE and EC (p=0.012). Similarly, comparison of EH and EC revealed a statistically significant difference (p = 0.014) (Table 2; Figure 9).

Of the 18 EC cases, 12 were high-grade, and six were low-grade. The mean total positivity for Ki-67 expression in high-grade EC was 71.74 (SD 21.26), whereas in low-grade EC it was 37.00 (SD 6.51). The mean strong positivity for Ki-67 expression in high-grade EC was 65.88 (SD 23.21), whereas in low-grade EC it was 31.26 (SD 6.80). The mean weak positivity of Ki-67 expression was 5.78 (SD 4.15) in low-grade EC and 5.35 (SD 5.18) in high-grade EC.

Total positivity and strong positivity of Ki-67 expression were higher in high-grade EC compared to low-grade EC, with statistically significant p values of 0.02 and 0.01, respectively (Table 3).

**Table 3:** Comparison of total, strong, and weak Ki-67 positivity between low-grade and high-grade EC ^*^P-value <0.05 is considered significant. EC: endometrial carcinoma

S. no	Statistical parameters	Total positivity	Strong positivity	Weak positivity
1.	Test statistics (Mann-Whitney test)	5.00	4.00	24.50
2.	P-value	0.02^*^	0.01^*^	0.29

## Discussion

In the present study, the maximum number of cases was observed in the 41-50 years age group, followed by the 51-60 years age group. Similar age distribution was reported in a study by Kumar KTAK et al. [13], with an age range of 42-70 years. In their study, EH without atypia and EH with atypia were observed in the 40-50-year age group. In the present study, EH without atypia was most common in the 30-50 years age group, and EH with atypia was more commonly noted in the 40-60 years age group. They also mentioned that all cases of EC occurred in individuals between the ages of 50 and 70 years. Similar findings were observed in the present study, with the maximum number of EC cases occurring in the 50-70 years age group.

Somani et al. [10] mentioned that the most common clinical presentations were menorrhagia and postmenopausal bleeding, amounting to 20 (33.34%) cases each. Our study findings correlate with these observations. In the present study, most patients complained of menorrhagia followed by abnormal uterine bleeding.

In a study by Manimaran A. et al. [4], cases of DPE were 15 (45.45%), EH without atypia were 16 (48.48%), and EH with atypia were two (6.06%). In the study conducted by Ozuysal et al. [14], cases of EH with atypia were 14 (15.73%), EH without atypia were 15 (16.86%), and EC were 30 (33.70%). In the present study, comparable findings were noted, with a relatively higher number of cases of EC 18 (26.47%) and EH with atypia 13 (19.12%).

In a study by Gharib et al. [9], it was reported that in EH without atypia, Ki-67 expression was lower than 40%, whereas in EH with atypia, it was 80%. They observed Ki-67 positivity in 100% of EC cases. A similar observation was noted in the present study, in which all EH with atypia and EC cases showed Ki-67 positivity. In a study by Shevra et al. [3] on normal, hyperplastic, and neoplastic endometrium, it was observed that in EH with atypia, Ki-67 expression was greater than 30%, and in EC, it was greater than 57%. Comparable results were found in the present study.

In one study, Ki-67 was reported to be a useful prognostic factor in stage I and stage II EC. These authors also reported that the optimal cut-off value of the Ki-67 labeling index for predicting EC recurrence was 38.0% [15]. Al-Nuiamy et al. [7] reported that the mean total Ki-67 positivity was highest in EC compared with EH. Similar findings were observed in our study. Manimaran A. et al. [4] reported that Ki-67 expression was higher in EH with atypia than in EH without atypia. Our study findings correlate with these findings.

Limitations of the study

In the present study, follow-up was not done; hence, prognosis and survival could not be assessed. A molecular study was not done, hence the risk factor could not be assessed in the present study. The number of cases of EC and EH was lower. Hence, extensive multicentric studies with a larger number of cases with follow-up may help in predicting the utility of Ki-67 in assessing the prognosis of EC.

## Conclusions

The total positivity for Ki-67 expression was highest in EC, followed by AEH. In the present study, Ki-67 expression in DPE was slightly higher compared to EH without atypia. This may be due to the smaller sample size in EH without atypia as compared to the DPE. Total Ki-67 expression was also higher in AEH than in EH without atypia. These findings suggest that Ki-67 is useful in differentiating DPE from EH without atypia, particularly when H&E-stained slides are difficult to interpret. In addition, it may facilitate the distinction between EH without atypia and AEH in diagnostically challenging cases. Hence, Ki-67 marker studies may be useful in endometrial lesions such as DPE, EH without atypia, and AEH. These findings further suggest that proliferative activity increases progressively from DPE to EC.

Ki-67 expression was significantly higher in high-grade EC than in low-grade EC, suggesting a role for Ki-67 as a prognostic indicator and biomarker of aggressiveness.

## References

[1] Siegel RL, Miller KD, Jemal A (2016). Cancer statistics, 2016. CA Cancer J Clin.

[2] Corr BR, Erickson BK, Barber EL, Fisher CM, Slomovitz B (2025). Advances in the management of endometrial cancer. BMJ.

[3] Shevra CR, Ghosh A, Kumar M (2015). Cyclin D1 and Ki-67 expression in normal, hyperplastic and neoplastic endometrium. J Postgrad Med.

[4] Manimaran A, Dongapure S (2024). Expression of Bcl-2 and Ki-67 in endometrial hyperplasia and disordered endometrium: a cross-sectional study. Natl J Lab Med.

[5] Ghalib Farhood R, Abd Ali Al-Humairi I (2022). Immunohistochemical study of Ki-67 in hyperplastic and endometrium carcinoma: a comparative study. Arch Razi Inst.

[6] Kurman Ellenson LH, Ronnett BM (2019). Blaustein’s pathology of the female genital tract. https://link.springer.com/referencework/10.1007/978-3-319-46334-6.

[7] Alnuaimy S, Al-Allaf L, Al-Omar Z (2021). Clarification of Ki67 expression in association with the histological picture of endometrium in cases with abnormal uterine bleeding in Nineveh Province. Ann Coll Med Mosul.

[8] Truskinovsky AM, Lifschitz-Mercer B, Czernobilsky B (2014). Hyperplasia and carcinoma in secretory endometrium: a diagnostic challenge. Int J Gynecol Pathol.

[9] Gharib F, Abd Elaziz Mohamed D, Amer BS (2020). Expression of L1CAM and Ki-67 in endometrial cancer of Egyptian females: clinical impact and survival. Tumori J.

[10] Somani A, Nimbargi R, Patil A, Patil A, Bharadwaj R (2024). BCL2 and Ki 67 expression in endometrial hyperplastic disorders: an observational study in a tertiary care centre. Trop J Pathol Microbiol.

[11] Yerushalmi R, Woods R, Ravdin PM, Hayes MM, Gelmon KA (2010). Ki67 in breast cancer: prognostic and predictive potential. Lancet Oncol.

[12] Paleari L, Rutigliani M, D’Ecclesiis O (2023). Exploring the prognostic and predictive roles of Ki-67 in endometrial cancer. Int J Transl Med.

[13] Kumar KTAK, Upadhyaya K, Cn RT (2023). Role of Ki 67 in normal endometrium, endometrial hyperplasia and endometrial carcinoma: a comparative study. J Chem Health Risks.

[14] Ozuysal S, Oztürk H, Bilgin T, Filiz G (2005). Expression of cyclin D1 in normal, hyperplastic and neoplastic endometrium and its correlation with Ki-67 and clinicopathological variables. Arch Gynecol Obstet.

[15] Jiang P, Jia M, Hu J, Huang Z, Deng Y, Lai L, Ding S, Hu Z (2020). Prognostic value of Ki67 in patients with stage 1-2 endometrial cancer: validation of the cut-off value of Ki67 as a predictive factor. Onco Targets Ther.

